# Application of microRNA in Human Osteoporosis and Fragility Fracture: A Systemic Review of Literatures

**DOI:** 10.3390/ijms22105232

**Published:** 2021-05-15

**Authors:** Yen-Zung Wu, Hsuan-Ti Huang, Tsung-Lin Cheng, Yen-Mou Lu, Sung-Yen Lin, Cheng-Jung Ho, Tien-Ching Lee, Chia-Hao Hsu, Peng-Ju Huang, Han Hsiang Huang, Jhong-You Li, Yu-De Su, Shih-Chieh Chen, Lin Kang, Chung-Hwan Chen

**Affiliations:** 1 Orthopaedic Research Center, Kaohsiung Medical University, Kaohsiung 80701, Taiwan; i54011301@ncku.edu.tw (Y.-Z.W.); hthuang@kmu.edu.tw (H.-T.H.); junglecc@kmu.edu.tw (T.-L.C.); yemolu@kmu.edu.tw (Y.-M.L.); sungyenlin@kmu.edu.tw (S.-Y.L.); u105801010@kmu.edu.tw (C.-J.H.); u103800014@kmu.edu.tw (T.-C.L.); u106801002@kmu.edu.tw (C.-H.H.); roger01@ms4.hinet.net (P.-J.H.); u109508016@kmu.edu.tw (J.-Y.L.); u9401112@kmu.edu.tw (Y.-D.S.); 2 Department of Orthopedics, Kaohsiung Medical University Hospital, Kaohsiung Medical University, Kaohsiung 80701, Taiwan; 3 Regeneration Medicine and Cell Therapy Research Center, Kaohsiung Medical University, Kaohsiung 80701, Taiwan; 4 Musculoskeletal Regeneration Research Center, Kaohsiung Medical University, Kaohsiung 80701, Taiwan; 5 Department of Orthopedics, College of Medicine, Kaohsiung Medical University, Kaohsiung 80701, Taiwan; 6 Department of Orthopedics, Kaohsiung Municipal Ta-Tung Hospital, Kaohsiung 80145, Taiwan; 7 Department of Physiology, College of Medicine, Kaohsiung Medical University, Kaohsiung 80701, Taiwan; 8 Department of Veterinary Medicine, National Chiayi University, Chiayi 60004, Taiwan; hhuang@mail.ncyu.edu.tw; 9 Department of Orthopedics, Kaohsiung Municipal Hsiao-Kang Hospital, Kaohsiung Medical University, Kaohsiung 812, Taiwan; 10 Department of Healthcare Administration and Medical Informatics, Kaohsiung Medical University, Kaohsiung 80701, Taiwan; u108705012@kmu.edu.tw; 11 Department of Medical Records, Kaohsiung Medical University Hospital, Kaohsiung Medical University, Kaohsiung 80701, Taiwan; 12 Department of Obstetrics and Gynecology, National Cheng Kung University Hospital, College of Medicine, National Cheng Kung University, Tainan 701, Taiwan; 13 Institute of Medical Science and Technology, National Sun Yat-Sen University, Kaohsiung 80420, Taiwan; 14 Graduate Institute of Animal Vaccine Technology, College of Veterinary Medicine, National Pingtung University of Science and Technology, Pingtung 912301, Taiwan

**Keywords:** blood sample, fragility fracture, microRNA, osteoporosis, osteoblast differentiation, osteoclast differentiation

## Abstract

MicroRNAs (miRNAs) could serve as ideal entry points to the deregulated pathways in osteoporosis due to their relatively simple upstream and downstream relationships with other molecules in the signaling cascades. Our study aimed to give a comprehensive review of the already identified miRNAs in osteoporosis from human blood samples and provide useful information for their clinical application. A systematic literature search for relevant studies was conducted in the Pubmed database from inception to December 2020. We set two essential inclusion criteria: human blood sampling and design of controlled studies. We sorted the results of analysis on human blood samples according to the study settings and compiled the most promising miRNAs with analyzed diagnostic values. Furthermore, in vitro and in vivo evidence for the mechanisms of the identified miRNAs was also illustrated. Based on both diagnostic value and evidence of mechanism from in vitro and in vivo experiments, miR-23b-3p, miR-140-3p, miR-300, miR-155-5p, miR-208a-3p, and miR-637 were preferred candidates in diagnostic panels and as therapeutic agents. Further studies are needed to build sound foundations for the clinical usage of miRNAs in osteoporosis.

## 1. Introduction

Osteoporosis affects more than ten million Americans over age 50, leading to an estimated two million incident fractures and a total cost of $19 billion per year in the U.S. [1,2]. Often manifested as a silent disease, osteoporosis remains underdiagnosed and thus undertreated [1]. Currently, dual-energy X-ray absorptiometry (DXA) is still the most widely used technique for bone mineral density (BMD) assessment. However, the measurement by DXA could not provide enough clues to the underlying mechanisms resulting in osteoporosis. MiRNAs, in contrast, are widely studied in recent years and its measurement may provide vital information of the epigenetic environment [3,4].

MiRNAs are small non-coding endogenous RNA molecules (around 19–25 nucleotides in length), which regulate post-transcriptional gene expression [5]. The biogenesis of miRNAs is regulated at multiple levels, from initial miRNA transcription and processing by RNase Drosha in the nucleus, further processing by RNase Dicer and modification in the cytoplasm, loading onto the RNA-induced silencing complex (RISC) as a functional unit, to finally RNA decay [6]. After incorporation into the RISC, miRNAs usually act as a guide that base-pair with the 3′ untranslated region (3′UTR) of their target mRNAs, and then the double-stranded miRNA-mRNA complex induces the subsequent translational repression and mRNA degradation, thereby silencing the target mRNAs [7]. The alteration of miRNA expression could therefore passively suggest the mechanism of a disease, and it is also possible to control the disease actively by producing a change to the regulation of miRNA.

It is challenging to measure the expression level of intracellular miRNAs. Instead, extracellular miRNAs are easily detectable, and the measurement is meaningful since they may serve as mediators for intercellular communication. Studies indicate that extracellular miRNAs come from three main routes [8,9]. First, they could be transported by extracellular vesicles, either by exocytosis or direct budding of the plasma membrane. Second, they could bind to specific proteins such as lipoprotein and ribonucleoproteins, then secreted in the form of protein-miRNA complexes. In addition, they may also come from damaged or dead cells.

In a previous study, the majority (>80%) of the studied miRNAs is found in various tissues, but there are still some miRNAs found to be tissue-specific [8]. In the domain of osteoporosis, we hope to identify the miRNAs involved in the pathways of bone homeostasis such as transforming growth factor-beta (TGF-β)/bone morphogenic protein (BMP) signaling for osteoblast differentiation and the osteoprotegerin (OPG)/receptor activator of nuclear factor Kappa-B ligand (RANKL)/RANK pathway for osteoclast differentiation [10,11]. In addition to identification, miRNAs could serve as ideal entry points to explore the full picture of deregulated gene expression in osteoporosis and further highlight the major dysfunctional pathways according to the etiology.

As more and more researchers are devoted to studying miRNAs in osteoporosis, evidence from in vitro, in vivo experiments and clinical trials is getting abundant. Reviews on this topic published in recent years started to focus on specific issues rather than giving a general description on the function of miRNAs. For, instance, a meta-analysis published in 2019 examined the miRNAs as potential biomarkers for postmenopausal osteoporotic patients, and a recent review particularly described the strength and weakness in practical usage of miRNAs [3,12]. For a step toward clinical applications, we focused on the studies that included human blood samples and demonstrated the research findings step by step. Demonstrating the results from the evidence of direct increased or decreased expression of the miRNAs in human blood samples, the in vitro evidence for the possible mechanisms of the above miRNAs, to the evidence that confirms the pathways in animal models, our study aims to target the miRNAs with top priorities for further clinical studies or usage.

## 2. Materials

### 2.1. Searching Strategy

We systematically searched the Pubmed database to screen relevant articles from inception to December 2020, without restriction to language. The following key terms with Boolean operators were adopted to search articles: (“micro RNA” OR miRNA OR miR) AND (osteoblastogenesis OR “osteoblast differentiation” OR osteoclastogenesis OR “osteoclast differentiation” OR osteoporosis OR osteoporotic) AND (serum OR blood sample OR circulating) AND (patient OR participant). Titles and abstracts of all the identified articles in the database were screened for potential studies. Next, full texts of the potential studies were further examined. The screening procedure was performed independently by two reviewers (Y.Z.W. and C.H.C.). Any discrepancy was solved by discussion until the two reviewers reached a consensus.

### 2.2. Inclusion and Exclusion Criteria

Studies were included if the following criteria were met: (1) clinical trials that examined miRNA in the field of osteoporosis; (2) a study population with at least a group of osteoporotic patients and a group of controls, without restriction to gender; (3) diagnosis of osteoporosis confirmed by DXA or clinical low-impact fracture (fragility fracture); (4) collected samples with at least human blood samples. The exclusion criteria were as follows: (1) studies that included other fields such as osteonecrosis; (2) a control group with other known bone diseases such as osteoarthritis.

### 2.3. Study Selection

**Figure 1:**
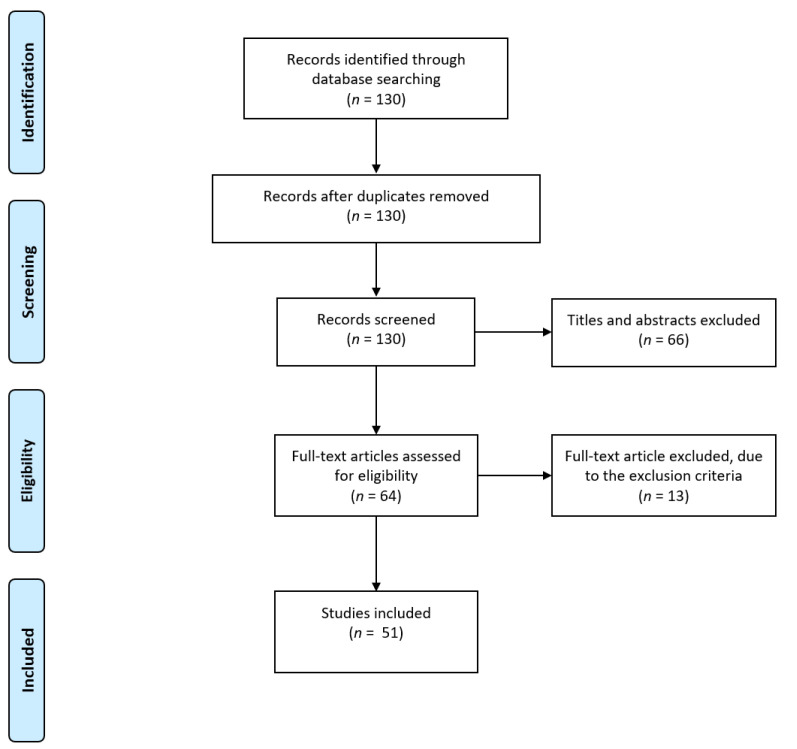
Flow diagram of study selection.

The flow diagram of the selecting process is shown in Figure 1. Initially, 130 relevant articles were identified from the Pubmed database. Titles and abstracts of all the articles were screened, and 64 of them were considered as eligible. We further reviewed the full texts of the remaining studies, of which 51 studies met our inclusion criteria and were included in our study.

### 2.4. Study Characteristics

The main characteristics of the included studies are shown in Table 1. A least 3834 patients were included in this study (four studies did not provide sample size), and 89% (2475/2784) of the patients were female according to the available data. Mean age of the participants ranged from 34.0 to 85.8, which varied widely due to the study setting. Extracted outcomes included the analysis on human blood samples, as well as the results of in vitro and in vivo experiments.

## 3. Serum miRNAs as Clinical Potential Biomarkers for Human Osteoporosis

To be a useful clinical examination, the following three elements are usually required: availability (i.e., convenience), appropriateness (i.e., fulfilling indications), and diagnosability. For availability, we set human blood sampling as an essential inclusion criterion for its convenience in clinical sampling compared to bone biopsy. For appropriateness, we arranged the studied miRNAs according to their study settings. For diagnosability, diagnostic values of specific analyzed miRNAs were presented, and we recommended usage of multiple miRNAs in combination for a more comprehensive clinical judgement.

In our included studies, up to 851 miRNAs were detected in a single study for screening. Most of the studies made the detection of potential miRNAs in one round, while five studies carried out a discovery analysis with a small sample size using microarray or quantitative real time polymerase chain reaction (qPCR) to recognize the most deregulated miRNAs at first, followed by a validation analysis that tested the potential miRNAs with a larger sample size for confirmation [14,15,16,21,29]. Some studies further assessed the diagnostic values of selected miRNAs, and a receiver operating characteristic (ROC) curve analysis was often performed.

All included studies had a group of osteoporotic patients and a control group, but several distinct additional controlled variables were adopted by individual study, including menopause, fracture, and advanced age. The settings were designed by the researchers to identify the target miRNAs precisely under specific conditions, but we should still interpret the results with caution for at least the following two reasons. One is that circulating miRNAs in the serum are the combination of the physiological and pathological actions from all kinds of organs and tissues in the body. The other one is that the presentation may vary with different stages of the pathological actions, such as a compensatory effect in an unbalance homeostasis (not the primary cause). Trying not to over-interpret the results of a single miRNA, we recommended the usage of a combination of identified miRNAs for diagnosis, which was often referred to as miRNA signature, and tissue-specific miRNAs were preferred.

**Figure 2:**
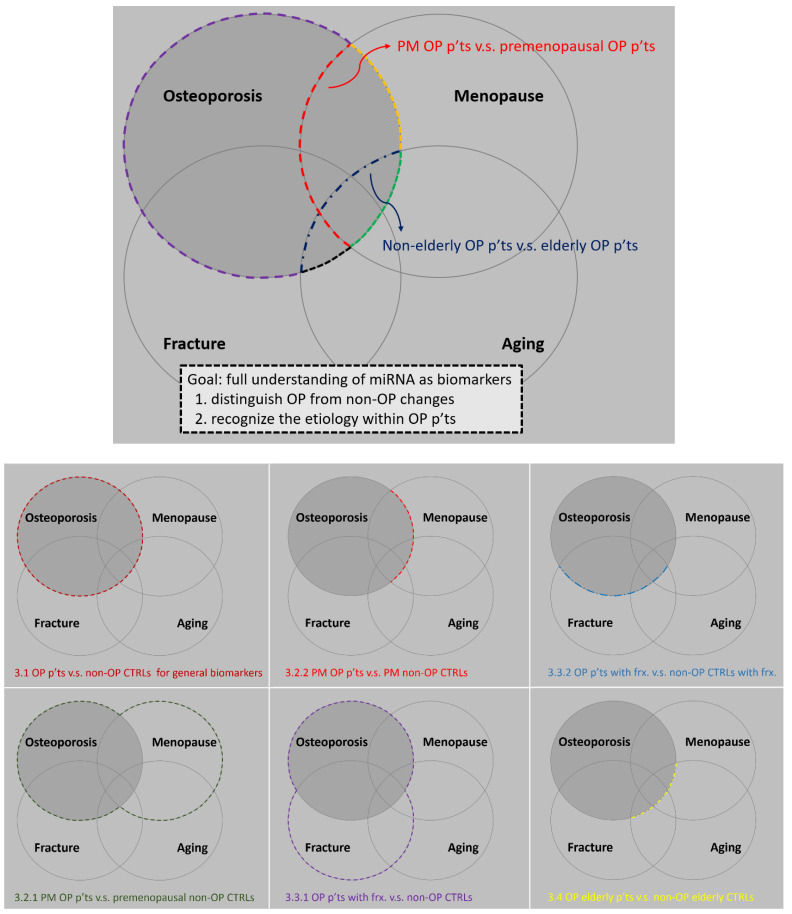
Schematic diagram of the de-regulated miRNAs by clinical settings. Take the red curve, for instance; it demarcates the difference in miRNA expression between postmenopausal and premenopausal groups in osteoporotic women; that is, the red curve represents the changes by estrogen deficiency leading to osteoporosis.

We used Venn diagrams to demonstrate the relationships of the main independent variables (menopause, fracture, aging) and dependent variable (osteoporosis) in Figure 2. The border between two areas is representative of the difference between them. The ultimate goal is to figure out the:

(1) outer border of osteoporosis: distinguish osteoporotic patients from normal people who have a similar risk factor

(2) inner demarcation within osteoporosis: recognize the etiologies among osteoporotic patients

### 3.1. General Biomarkers for Distinguishing Osteoporotic Patients from Non-Osteoporotic Controls

The following two study settings were categorized in this group. Without additional controlled variables, the identified miRNAs in this group could hardly provide clues for specific etiology.

- Comparison between osteoporotic patients and controls [16,20,22,24,27,29,31,32,34,35,36,40,41,44,45,47,50,51,52,53,55,59,61,63]
- Comparison between osteoporotic patients, osteopenia patients, and controls [15,18,56,57]

Thirty-seven potential miRNAs with significant up-regulation and 19 with significant down-regulation in osteoporotic patients compared to the control group are listed in Table 2. Among the identified miRNAs, five miRNAs (miR-21-5p, miR-24-3p, miR-93-5p, miR-100-5p, miR-125b-5p) were found also significantly up-regulated in bone tissue from osteoporotic patients compared to controls [22]. Some of the studies further analyzed the correlation of the miRNA regulation with bone parameters, which are also illustrated in Table 2.

It is noteworthy that conflicting results of miR-21 regulation were found by different studies, and it is the same case in the clinical setting comparing postmenopausal osteoporotic patients to postmenopausal non-osteoporotic controls. Further studies are needed to investigate its regulation.

### 3.2. miRNAs That Are Potentially Associated with Estrogen

Estrogen is a well-known important regulator in female osteoporosis, and the interactions between miRNAs and estrogen or its receptor have been studied extensively. For instance, miR-18a, miR-22, and miR-206 are found to target estrogen receptor (ER) α, and 17β-estradiol also regulates the expression of various miRNAs by several ER-mediated signaling pathways [64].

#### 3.2.1. Studies That Aim to Accentuate the Role of Estrogen

- Comparison between postmenopausal osteoporotic patients and controls [25,28,37,39,46,60]
- Comparison between postmenopausal osteoporotic patients and premenopausal controls [21,48]

There were six significantly up-regulated and five significantly down-regulated miRNAs in osteoporotic patients compared to the control group identified and listed in Table 3. To confirm the relationship between the identified miRNAs and bone metabolism further, miR-28, miR-373, and miR-101 were also found to be down-regulated in the bone tissue by qPCR analysis, and the expression of miR-133a in the serum was found to be negatively correlated with lumbar BMD [25,37].

#### 3.2.2. Studies That Aim to Attenuate the Influence of Estrogen Itself

The design of this setting contains at least two purposes. One is to offset the regulation of miRNAs influenced by menopause; the other one is to identify the most common pathogenesis in the postmenopausal women.

- Comparison between postmenopausal osteoporotic patients and postmenopausal non-osteoporotic controls [13,15,17,33,42,43,58]

There were 12 miRNAs found to be significantly up-regulated and three miRNAs significantly down-regulated in osteoporotic patients compared to the control group. Tang et al. 2019 conducted an enzyme-linked immunosorbent assay (ELISA) to detect serum levels of Sfrp1 and TNF-α, and a similar trend with miR-144 was observed [43]. In Suarjana et al. 2019, analysis revealed that miR-21 was positively correlated with serum RANKL level and RANKL/OPG ratio, and it also negatively correlated with TGF-β1, OPG, and BMD in the postmenopausal osteoporotic group. They further carried out a linear regression analysis, and the following relationship was documented [42].

BMD = 1.373 − 0.085 × Ln. miR-21 − 0.176 × Log_10_RANKL (1)

### 3.3. miRNAs That Are Potentially Associated with Fracture Healing

After a fracture, the healing process includes inflammation, bone formation, and bone remodeling. The expression of miRNAs is found to altered significantly during the healing process [65]. The identified fracture-related miRNAs with different expression level between osteoporotic patients and the control group were listed in Table 4.

#### 3.3.1. Study That Aims to Accentuate the Role of Sustained Fractures

- Comparison between osteoporotic patients with sustained low-traumatic fractures and controls [19]

Nineteen miRNAs were found significantly regulated in osteoporotic patients (male, premenopausal, and postmenopausal subgroups) compared to the control group. Among the identified miRNAs, the ROC curve analysis showed that eight miRNAs (miR-140–5p, miR-152–3p, miR-30e-5p, miR-324–3p, miR-335–3p, miR-19a-3p, miR-19b-3p, miR-550a-3p) were potential candidates (area under curve (AUC) values > 0.9) as biomarkers for the tendency of low-traumatic fractures. Moreover, miR-93-5p and miR-324-3p were also significantly correlated with lumbar spine areal BMD [19].

#### 3.3.2. Studies That Aim to Attenuate the Osteogenic Effect After a Recent Fracture

The design of this setting has two basic considerations. One is to cancel out the regulation of miRNAs by a common osteogenic effect after a recent fracture; the other one is to distinguish the reactions to fractures in osteoporotic patients from the controls.

- Comparison between osteoporotic patients with fracture and non-osteoporotic controls with fracture [14,30,38,54]

There were 14 miRNAs found to be significantly up-regulated and one significantly down-regulated in the osteoporotic groups compared to control groups, and they were listed in Table 5. Seeliger et al. 2014 and Wang et al. 2018 further tested the identified miRNAs in bone tissues, and the results showed that an up-regulation of miR-21, miR-23a, miR-24, miR-24-3p, miR-27a-3p, miR-100, miR-122a, and miR-125b was consistently observed in both blood samples and bone tissues [14,30].

### 3.4. miRNAs That Potentially Associate with Osteoporosis in Elderly People

Aging is one of the important risk factors resulting in osteoporosis. From the cellular aspects, cell senescence leads to telomere shortening, a change in gene expression, epigenetic regulators, and protein processing [66]. Many miRNAs have been identified in other age-related diseases. MiR-146, miR-155, miR-21, and miR-126, for example, are found to be helpful for differentiating patients with cognitive impairment from the age-matched controls [67].

This setting aimed to offset the ubiquitous influence by advanced age; meanwhile, the difference in the miRNA expressions may represent the common deregulated pathways in elderly people.

- Comparison between elderly osteoporotic patients and elderly controls [26].

MiR-96 and miR-107 were up-regulated in osteoporotic patients compared with healthy controls, and the serum level of miR-96 was also significantly higher in the elderly group compared to the young group [23,26].

### 3.5. miRNAs with High Diagnostic Value for Osteoporosis

ROC analysis was performed in nine studies for assessing the diagnostic value of the identified miRNAs with the greatest potential as osteoporotic biomarkers. The result is shown in Table 6.

Besides using a single miRNA as a biomarker for osteoporosis, the results of Mandourah et al. 2018 suggested that miR-122-5p and miR-4516 be used together to increase the diagnostic value [27]. In Kocijan et al. 2016, the results of a multivariate model revealed that the highest predictive power was reached by using a combination of miR-155–5p, miR-181c-5p, miR-203a, miR-330–3p, and miR-942–5p (AUC: 0.97) [19].

In Shuai et al. 2020, two indices were developed to distinguish osteoporosis patients from healthy controls, shown as follows [56]:

- Index 1 = −0.394 + (0.105 × miR-30c-2-3p) + (−1.022 × miR-199a-5p) + (−0.078 × miR-424-5 p) + (− 0.046 × miR-497-5p) + (0.089 × miR-877-3p) (AUC: 0.86)
- Index 2 = (miR-30c-2-3p + miR-877-3p) − (miR-199a-5p + miR-424-5p) (AUC: 0.77)

## 4. Mechanisms of the Identified miRNAs

To study the mechanism of the potential miRNAs identified by blood sample analysis, the role of the miRNAs was explored step by step.

In an in vitro experiment, several types of cells were commonly used. Mesenchymal stem cells (MSCs) such as bone marrow mesenchymal stem cells (BMSCs) or adipose-derived mesenchymal stem cells (ADSCs) were cultured for studying the differentiation of osteoblast, and peripheral blood mesenchymal cells (PBMCs) for osteoclast differentiation. The first step was to determine whether either the osteoblastic or osteoclastic pathway was the selected miRNA involved. This was usually established by transfecting the cells with miRNA mimics or inhibitors to alter expression of the miRNA, followed by detecting the activities of the cells related to bone metabolism (ex. alizarin red stain and alkaline phosphatase (ALP) staining) and the expression of specific transcriptional regulators (dual-luciferase reporter gene assay) or proteins (western blot analysis). Next, the target of the selected miRNA was usually predicted by tools such as Targetscan, miRanda or miRWalk software. The final step was to verify the hypothesized pathway. It was usually performed by transfecting the cells with miRNA mimics or inhibitors, and co-transfection with siRNA for the target gene, followed by detecting the expression of involved molecules and making a deduction from the results.

In an in vivo experiment, animals were usually injected with miRNA mimics or inhibitors, and tools such as micro-CT and many bone parameters were used for assessing the difference in bone architecture in the experiment group compared to the control group.

### 4.1. miRNAs Involved in Osteoblastogenesis

**Figure 3:**
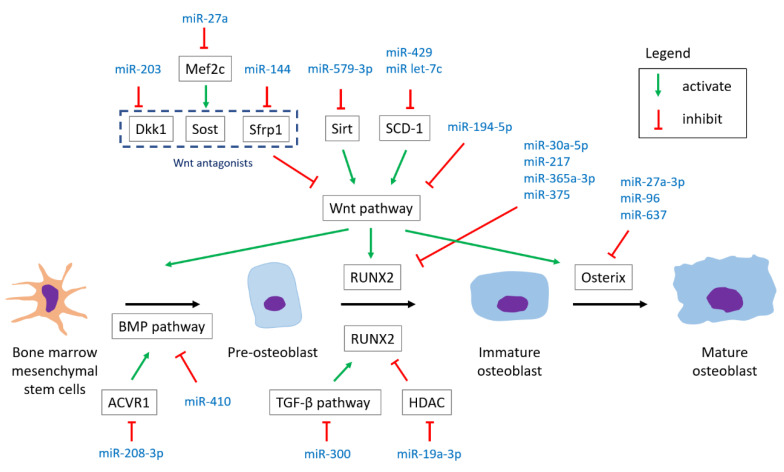
The relationship between the miRNAs and the involved pathways in osteoblastogenesis. Dkk1, Dickkopf 1; Sost, Sclerostin; Sfrp, secreted frizzled-related protein; Sirt, sirtuin; SCD-1, stearoyl CoA desaturase; ACVR1, activin A receptor type I; HDAC, histone deacetylase.

The relationship between the miRNAs and the involved reactions is illustrated in Figure 3 [68] and Table 7. We demonstrated the miRNAs from the involvement in important signaling pathways to transcriptional regulators, and the promotive or inhibitive effects on osteoblastogenesis were described after every single miRNA.

#### 4.1.1. Wnt Pathway

Wnt proteins comprise a family of secreted glycoproteins that play a central role in osteoblast differentiation. When Wnt binds to its principal receptor (frizzled protein) and co-receptor, multiple intracellular signaling cascades are activated, including the canonical β-catenin-dependent pathway and noncanonical β-catenin-independent pathway. In the canonical β-catenin-dependent pathway, β-catenin translocates into the nucleus and interacts with several transcriptional factors, thereby stimulating gene expression. Besides, Wnt signaling is regulated by various antagonists, such as Dickkopf 1 (Dkk1), Dkk2, and Sclerostin (Sost) proteins; secreted frizzled-related protein 1 (Sfrp1) inhibits the formation of Wnt-frizzled complexes by directly binding to the Wnt ligand [73].

In our included studies, there were eight miRNAs found to be involved in the regulation of Wnt signaling pathways.

- MiR-194-5p targets Wnt 5a, suppressing osteoblast differentiation. An in vivo study by micro-CT analysis disclosed that adult mice injected with miR-194-5p over femoral bone marrow had significantly decreased bone parameters (BMD and BV/TV) over the femur compared to controls three months later [53].
- MiR-144 and miR-203 target Wnt antagonists (Sfrp1 and DKK1, respectively), promoting osteoblast differentiation [28,43]. Besides, ovariectomized rats injected with antagomir-203 had decreased BMD over tibia and bone volume parameters compared to the control group injected with mutant antagomiR-203 six weeks after the injection [28].
- MiR-27 targets Mef2c which activates Sost protein, promoting osteoblast differentiation [21,74]. An in vivo study revealed that antagomiR-27-treated mice had up-regulated expression of the Mef2c protein and lower bone parameters (BMD and BV/TV) compared to the controls [21].
- MiR-429 and miR let-7c both target stearoyl CoA desaturase (SCD-1, an enzyme that activates the Wnt protein), inhibiting osteoblast differentiation [48,53].
- MiR-579-3p targets Sirt which deacetylates β-catenin and promotes Wnt signaling, inhibiting osteoblast differentiation [40,75].
- MiR-23b-3p targets MRC2 and is found to suppress Wnt signaling, inhibiting osteoblast differentiation. Although a relationship between MRC2 and Wnt signaling remained unclear, an in vivo study showed that OVX mice injected with the lenti-miR-23b-3p inhibitor had improved bone parameters [69].

#### 4.1.2. TGF-β Pathway

TGF-β is secreted and stored in the extracellular matrix. Activated TGF-β binds to the tetrameric receptor complex, which is composed of a TGF-β type I receptor (TβRI or ALK5) and type II receptor (TβRII). Downstream signaling included canonical (Smad-dependent) and non-canonical (non-Smad-dependent) pathways. In the canonical pathway, R-Smad (Smad2 or 3) form complexes with Smad4 and regulate gene expression. Smad7, as a regulatory molecule, competes against Smad2 or 3 for binding to Smad4 [10,76].

In our included studies, there was one miRNA found to be involved in the regulation of the TGF-β signaling pathway.

- MiR-300 targets Smad3, inhibiting osteogenic differentiation. Moreover, micro-CT for evaluation in a rat model showed that miR-300 injections led to lower bone parameters (BMD and BV/TV) compared to sham and negative control groups [52].

#### 4.1.3. BMP Pathway

BMP is vital for embryonic skeletal development and bone homeostasis after birth. When BMP binds to its ligands, type II receptors will form a complex with type I receptors, leading to transphosphorylation of the type I receptors and signal transduction. Most BMPs activate the canonical BMP pathway (Smad-dependent), and BMP-2, 4, 5, 6, 7, and 9 are found to induce osteoblast differentiation and bone formation actively [10,76].

In our included studies, there was one miRNA involved in the regulation of the BMP pathway.

- MiR-410 targets BMP-2, inhibiting osteoblast differentiation. Besides, the up-regulation of miR-410 was found in both postmenopausal osteoporotic patients compared to healthy controls and in the OVX mice group compared to the sham group [46].

#### 4.1.4. Common Transcriptional Pathway

##### Runt-Related Transcription Factor 2 (RUNX2)

RUNX2 is an essential transcriptional factor in the osteoblast differentiation. The effect of RUNX2 depends on its interaction with other DNA sequences or proteins that may bind to the various domains of RUNX2 [68]. Studies revealed that Runx2 could up-regulate the expression of genes encoding bone matrix proteins, such as Col1a1, Spp1, Ibsp, bone gamma-carboxyglutamate protein (Bglap), and Fn1 [77].

In our included studies, four miRNAs were found to target RUNX2.

- MiR-30a-5p, miR-217, miR-365a-3p, and miR-375 target RUNX2, inhibiting osteogenic differentiation [34,36,44,45,50]. Moreover, miR-30a-5p is found to be involved in the XIXT/miR-30a-5p/RUNX2 axis; miR-217 is involved in both the TERC/miR-217/RUNX2 and circ-VANGL1/miR-217/RUNX2 axes [44,45,50].

##### Osterix

Osterix, also known as Sp7, is a transcriptional factor specific for osteoblast. It plays an important role in regulating the gene expression during differentiation of the pre-osteoblast into mature osteoblast. On the other hand, it was regulated by the BMP2 signaling pathway and insulin-like-growth-factor (IGF) pathway. The BMP2/Smad pathway activates RUNX2, which then activates the expression of osterix, whereas the IGF pathway activates osterix in a RUNX2-independent manner [78].

In our included study, there were three miRNAs found to target osterix.

- MiR-27a-3p, miR-96, and miR-637 target osterix, inhibiting osteogenic differentiation [26,59]. The experiment of a mice model showed that repetitively agomiR-96-injected young mice had significantly decreased BMD compared with vehicle-treated mice, and aged mice treated with antagomir-96 had higher bone strength compared to controls [26]. In addition, the signal transducer and activator of transcription 3 (Stat3) was found to be a pseudo-target of miR-637 by biological experiments [70].

#### 4.1.5. Other Reactions in Osteogenic Differentiation

##### Histone Deacetylase (HDAC)

HDACs are the component of transcriptional co-repressors complexes that regulate gene expression. HDAC4 is regulated by PTH, and the HDAC4 inhibitor may facilitate osteoblast differentiation [79,80].

- MiR-19a-3p targets HDAC4, promoting osteogenic differentiation [32].

##### Phosphatase and Tensin Homolog (PTEN)/Phosphoinositide 3-Kinases (PI3K)/AKT Signaling Pathway

AKT is inhibited in osteoblastic cells due to the abundance of PTEN within it. Although the PTEN/PI3K/AKT pathway is not a dominant signaling pathway in osteoblastic differentiation, inhibition of PTEN activity leads to increased AKT activation and subsequent cell proliferation [81].

- MiR-19b targets PTEN, promoting osteogenic differentiation. Moreover, bone parameters including BMD, bone volume, and trabecular number were significantly higher in ovariectomized mice injected with agomiR-19b than those of the negative control group [57].

##### ATF3

Activating transcription factor 3 (ATF3) is a transcriptional factor belonging to the ATF/cAMP response element-binding protein (CREB) family. ATF3 expression could be up-regulated by TNF- α through the JNK signaling pathway, and overexpression of ATF3 would in turn inhibit osteoblast differentiation [82].

- MiR-27a-3p targets ATF3, promoting osteogenic differentiation [35].

##### Glutaminase (GLS)

Glutamine could serve as a material in protein synthesis and also an energy source. Our included study showed that the uptake of L-glutamine increased with time during osteogenic differentiation induction at 7 and 14 days, suggesting that glutamine plays an important role in the differentiation [41].

- MiR-200a-3p targets glutaminase, inhibiting osteogenic differentiation [41]

##### Activin A Receptor Type I (ACVR1)

A study found that ACVR1-null mice had decreased expression levels of Wnt inhibitors Sost and Dkk1; canonical Wnt signaling was then increased and facilitated osteogenic differentiation. A hypothesis of the BMP7-ACVR1-SOST/DKK1 axis in osteoblasts was then proposed [83]. However, our included study showed opposite results. ACVR1 was found to correlate positively with BMP2, and downregulation of ACVR1 therefore led to suppression of osteoblastogenesis [71].

- MiR-208a-3p targets ACVR1, inhibiting osteogenic differentiation. An in vivo study revealed that hip-limb-unloading (HLU) mice treated with antagomiR-208a-3p had higher parameters in bone formation and trabecular microarchitecture compared to the HLU control group [71].

##### Proprotein Convertase Subtilisin/Kexin Type 5 (PCSK5)

PCSK belongs to a family of subtilisin-like serine proteinases and activates various precursor proteins and peptides. PCSK5-knockout mice have VACTERL (vertebral, anal, cardiac, trachea-esophageal, radius or renal, limb) syndrome-like malformations, and a recent study indicates that PCSK5 is expressed in mice osteoblast with osteopontin (OPN) as one of its substrates [84,85].

- MiR-338-3p targets PCSK5, inhibiting osteogenic differentiation [72].

### 4.2. miRNAs Involved in Osteoclastogenesis

**Figure 4:**
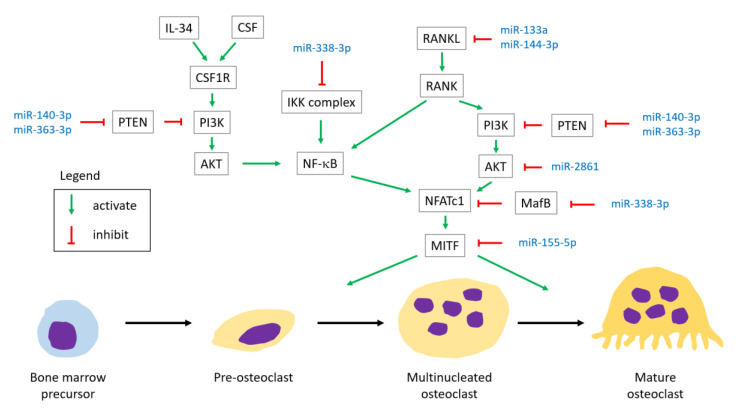
The relationship between the miRNAs and the involved pathways in osteoclastogenesis. IL-34, interleukin-34; CSF, colony stimulating factor; IKK, inhibitor of nuclear factor kappa-κ kinase; NFATc1, nuclear factor of activated T cells 1; MITF, microphthalmia associated transcription factor.

The relationship between the miRNAs and the involved reactions is illustrated in Figure 4 [11] and Table 8. To assess the activity of osteoclast, tartrate-resistant acid phosphatase (TRAP) staining is often used in the studies.

#### 4.2.1. RANK

RANKL is a well-known important factor for osteoclast differentiation. It binds to RANK and in turn induces recruitment of the tumor-necrosis-factor-receptor-associated factor 6 (TRAF6) protein, which activates downstream signaling pathways. NF-κB signaling was then activated, which is essential for osteoclast differentiation [89]. Nevertheless, OPG secreted by osteoblasts and other cells could bind to RANKL to prevent osteoclast formation, and MafB is a regulatory molecule that inhibits the RANKL pathway [90,91].

In our included study, there were four miRNAs found to be involved in the RANKL signaling pathway.

- MiR-144-3p targets RANK, inhibiting osteoclast differentiation [30].
- MiR-133a overexpression promotes RANKL-induced osteoclast differentiation. An in vivo study using a rat model showed that a significant decrease of osteoclastogenesis-related factors (M-CSF, RANKL, TNF-α, IL-1α, and CTX-I) was observed in OVX rats with a miR-133a knockdown compared to the controls [25].
- MiR-21 was found to correlate positively with RANKL level and the RANKL/OPG ratio, and correlate negatively with TGF-β1 and OPG by analysis of blood samples [42].
- MiR-338-3p targets MafB, promoting osteoclast differentiation [86].

#### 4.2.2. Colony Stimulating Factor-1 Receptor (CSF1R)

CSF1R, a type III receptor tyrosine kinase, plays an important role in the differentiation of myeloid cells, cancer development, and progression of various diseases [92]. When the colony stimulating factor (CSF) or interleukin-34 (IL-34) binds to CSF1R, it induces PI3K/AKT signaling [92,93]. Downstream transcription factors are in turn activated, including NFATc1 and NFkB, which are essential to osteoclast differentiation and function.

#### 4.2.3. Phosphatase and Tensin Homolog (PTEN)/Phosphoinositide 3-Kinases (PI3K)/AKT Signaling Pathway

The PTEN/PI3K/AKT pathway regulates various biological processes, such as cell metabolism, proliferation, growth, and vesicle trafficking [94]. PTEN inhibits PI3K signaling and is usually regarded as a tumor suppressor. A recent study showed that PTEN could suppress RANKL-induced signaling pathways, thereby inhibiting the activity of osteoclast [95].

In our included study, there were three miRNAs found to be involved in the PTEN/PI3K/AKT signaling pathway.

- MiR-140-3p and miR-363-3p target PTEN, promoting osteoclast differentiation [38,60].
- MiR-2861 targets AKT2, suppressing osteoclast differentiation [49].

#### 4.2.4. Others

##### Microphthalmia Associated Transcription Factor (MITF)

MITF and NFATc1 are important transcriptional factors in osteoclastogenesis, and they could be activated by RANKL signals. A recent study using a mice model demonstrates that semi-dominant mutation of the MITF gene results in arrest of osteoclastogenesis, and MITF may be downstream of NFATc1 in the RANKL pathway [96].

- MiR-155-5p targets MITF, inhibiting osteoclast differentiation [87].

##### Inhibitor of Nuclear Factor Kappa-κ Kinase Subunit β (IKKβ) Gene

The inhibitor of nuclear factor kappa-κ kinase (IKK) complex is crucial for the activation of classical NF-κB signaling pathways, which is usually induced by TNF, IL-1, or RANKL. In the classical NF-κB signaling pathway, two main components of the IKK complex are involved, IKKβ and IKKγ, which in turn degrade IκBα [97,98].

- MiR-338-3p targets IKKβ gene, inhibiting osteoclast differentiation [87].

## 5. miRNAs with Both Documented Diagnostic Values as Serum Biomarker for Osteoporosis and Identified Underlying Mechanisms

Based on the results of included studies, miRNAs with a verified diagnostic value as a serum biomarker and identified mechanisms were listed in Table 9 and regarded as preferred candidates for diagnostic panel or therapeutic agents. There are six miRNAs meeting the criteria without conflicting results: miR-23b-3p, miR-140-3p, miR-155-5p, miR-208a-3p, miR-300, and miR-637.

It is reasonable to use a combination of these miRNAs in a panel to aid the diagnosis of osteoporosis. However, the deregulation of a single miRNA is insufficient to ascertain the etiology of osteoporosis unless the miRNA is confirmed tissue-specific and not involved in other types of bone activities.

As potential therapeutic agents, the six miRNAs are preferred owing to their confirmed mechanisms and also their more noticeable deregulated expression level compared with all the other studied miRNAs. The higher the level of deregulated expression of miRNA, the more feasible the detection for diagnosis and monitoring after treatment.

It is noteworthy that Feurer et al. 2019, in a study with 682 women included, yield interesting findings. In the study, a number of selected miRNAs are found to be associated with fragility fractures, BTMs, BMD, and microarchitecture by comparing postmenopausal with premenopausal women, but the effect is negated after an age adjustment [99]. It is not disappointing because we can draw two inferences from the findings. First, the expression levels of some miRNAs are confirmed to be significantly different between postmenopausal and premenopausal groups despite the inability to recognize the etiology (estrogen or age). Second, the difference negated by the age adjustment means that the deregulated pathways are age-dependent, or, they may be related to both estrogen and age. The findings of the study underline the importance of study design (i.e., study setting), as the effort our study puts forth in part 3. If we interpret the results of studies with caution, every single finding could contribute to the final successful clinical usage.

## 6. Conclusions

With accumulating evidence verifying the association between deregulated miRNA expression and osteoporosis, it is necessary to analyze the collected data for further useful application in clinical settings. Previous studies mentioned the difficulty in integrating the results in clinical trials due to a lack of adequate controls or varied study settings despite the fact that there was already substantial experience and knowledge learned from in vitro or in vivo experiments [3,4]. Considering both the study setting and diagnostic value, our study demonstrated the most promising miRNAs as biomarkers for osteoporosis and evidence of mechanisms. We recommended miR-23b-3p, miR-140-3p, miR-300, miR-155-5p, miR-208a-3p, and miR-637 as preferred miRNAs for candidates in diagnostic panels and as therapeutic agents. In terms of the small sample size in each clinical study and little overlapping of the identified miRNAs among different studies, further studies are needed to build sound foundations and consensus for the clinical application of miRNAs.

## Figures and Tables

**Table 1:** Characteristics of the included studies.

Study/Reference	Main Studied miRNA	Sample Size	Patient Characteristics	Male: Female	Mean Age or Range of Age	Study Domain
Li et al. 2014 [13]	multiple	120	all PM female 40 OP p’ts v.s. 40 LBM p’ts v.s. 40 CTRLs	all female	57.5 (OP), 56.7 (LBM), 56.5 (CTRL)	human blood sample
Seeliger et al. 2014 [14]	multiple	63	all have a hip frx 33 OP p’ts v.s. 30 non-OP CTRLs	3: 60	NA	human blood sample human bone tissue sample
Meng et al. 2015 [15]	miR-194-5p	48 (discovery) 86 (validation)	25 OP p’ts v.s. 23 LBM p’ts 24 OP p’ts v.s. 30 LBM p’ts v.s. 32 CTRLs	all female	66.1 (OP), 64.7 (LBM) 64.0 (all 3 groups)	human blood sample
Weilner et al. 2015 [16]	multiple	14 (discovery) 23 (validation)	7 OP p’ts v.s. 7 CTRLs	all female	72.4 (OP), 71.0 (CTRL) 77.8 (OP), 81.5 (CTRL)	human blood sample in vitro: human ASC
Bedene et al. 2016 [17]	miR-148a	74	all PM female 17 OP p’ts v.s. 57 CTRLs	all female	62.0 (OP), 61.0 (CTRL)	human blood sample
Chen et al. 2016 [18]	multiple	36	all PM female 19 OP p’ts v.s. 7 LBM p’ts v.s. 10 CTRLs	all female	77.4 (OP), 72.86 (LBM), 51.89 (CTRL)	human blood sample animal blood sample
Kocijan et al. 2016 [19]	miR-29b-3p	75	36 p’ts with low-traumatic frx v.s. 39 CTRLs	20: 16 (OP) 23: 16 (CTRL)	46.6 (OP), 46.6 (CTRL)	human blood sample
Sun et al. 2016 [20]	miR-214	65	42 OP p’ts v.s. 23 CTRLs	NA	Men: 50–90 years old Woman: over 5 years of menopause	human blood sample in vitro: human PBMCs in vivo: mice model
You et al. 2016 [21]	miR-27a	155	81 OP PM p’ts v.s. 74 premenopausal CTRLs	all female	65.8 (OP), 43.3 (CTRL)	human blood sample in vitro: human MSC in vivo: mice model
Kelch et al. 2017 [22]	multiple	28	7 female OP p’ts v.s. 7 female CTRLs v.s. 7 male OP p’ts v.s. 7 male CTRLs	14: 14	81.9 (♀OP), 71.2 (♀CTRLs), 78.0 (♂OP), 68.6 (♂CTRLs)	human blood sample in vitro: osteoblast isolation & human PBMCs
Yavropoulou et al. 2017 [23]	miR-21-5p	100	all PM female 35 p’ts with LBM and vertebral frx v.s. 35 p’ts with LBM without frx v.s. 30 CTRLs	all female	68 (frx.), 71 (no frx.), 68 (CTRL)	human blood sample
Chen et al. 2018 [24]	multiple	18	9 OP p’ts v.s. 9 CTRLs	NA	69.2 (OP), 67.1 (CTRL)	human blood sample in vitro: human & mice osteoblast cells
Li et al. 2018 [25]	miR-133a	20	all PM female 10 OP p’ts v.s. 10 CTRLs	all female	59-80 (OP), 62–75 (CTRL)	human blood sample in vitro: human monocytic & murine macrophage cells in vivo: rats model
Liu et al. 2018 [26]	miR-96	80	20 PM OP p’t v.s. 20 premenopausal CTRLs; 20 elderly OP p’ts v.s. 20 elderly CTRLs	NA	45–60 65–80	human blood sample in vitro: human & mice MSC in vivo: mice model
Mandourah et al. 2018 [27]	miR-122-5p miR-4516	161	53 OP p’ts v.s. 78 LBM p’ts v.s. 30 CTRLs	30: 131	69.1 (OP), 65.9 (LBM), 67.0 (CTRL)	human blood sample
Qiao et al. 2018 [28]	miR-203	100	60 PM OP p’ts v.s. 40 CTRLs	NA	63.4 (OP), 59.3 (CTRL)	human blood sample in vitro: human MSC in vivo: rats model
Ramírez-Salazar et al. 2018 [29]	miR-140-3p miR-23b-3p	40 (discovery) 97 (validation)	20 OP p’ts v.s. 20 CTRLs 21 OP p’ts with frx. v.s. 26 OP p’ts without frx. v.s. 28 LBM p’ts v.s. 22 CTRLs	all female	73.8 (OP), 71.1 (CTRL) 82.5 (OP with frx.), 68.9 (OP without frx.), 64.3 (LBM), 60.5 (CTRL)	human blood sample
Wang et al. 2018 [30]	miR-144-3p	60	all have a hip frx 45 OP p’ts v.s. 15 non-OP CTRLs	NA	NA	human blood sample in vitro: human PBMC
Xia et al. 2018 [31]	miR-203	120	60 OP p’ts v.s. 60 CTRLs	all female	NA	human blood sample in vitro: rat MSC
Chen et al. 2019 (a) [32]	miR-19a-3p	84	42 OP p’ts v.s. 42 CTRLs	NA	NA	human blood sample in vitro: human MSC
Chen et al. 2019 (b) [33]	multiple	75	all PM female sacropenic p’t (1) v.s. sacropenic OP p’ts (15) v.s. OP p’t (46) v.s. CTRL (13)	all female	85.8 (sacropenic), 68.9 (sacropenic OP), 69.6 (OP), 68.9 (CTRL)	human blood sample
Cheng et al. 2019 [34]	miR-365a-3p	60	30 OP p’ts v.s. 30 CTRLs	NA	NA	human blood sample in vitro: human MSC
Fu et al. 2019 [35]	miR-27a-3p	40	20 OP p’ts v.s. 20 CTRLs	NA	NA	human blood sample in vitro: human MSC
Lei et al. 2019 [36]	miR-375	60	30 OP p’ts v.s. 30 CTRLs	NA	NA	human blood sample in vitro: human MSC
Li et al. 2019 (a) [37]	miR-373	40	20 PM OP p’ts v.s. 20 CTRLs	NA	NA	human blood sample human bone tissue sample in vitro: rats PBMC in vivo: rats model
Li et al. 2019 (b) [38]	miR-363-3p	12	all p’ts have a frx 6 OP p’ts v.s. 6 non-OP CTRLs	NA	NA	human blood sample in vitro: human PBMC, C2C12 cells
Lin et al. 2019 [39]	miR-338 cluster	30	15 PM OP p’ts v.s. 15 CTRLs	all female	58–68	human blood sample in vitro: mice PBMC in vivo: mice model
Luo et al. 2019 [40]	miR-579-3p	NA	OP p’ts v.s. CTRLs	NA	NA	human blood sample in vitro: human MSC
Lv et al. 2019 [41]	miR-200a-3p	60	30 OP p’ts v.s. 30 CTRLs	NA	NA	human blood sample in vitro: human MSC
Suarjana et al. 2019 [42]	miR-21	120	all PM hypoestrogenic female 60 OP p’ts v.s. 60 non-OP CTRLs	all female	62 (OP), 58.5 (CTRL)	human blood sample
Tang et al. 2019 [43]	miR-144	30	all PM female 15 OP p’ts v.s. 15 CTRLs	all female	54–64	human blood sample in vitro: rats MSC
Yang et al. 2019 [44]	miR-217	30	15 OP p’ts v.s. 15 CTRLs	NA	NA	human blood sample in vitro: human MSC
Zhang et al. 2019 (a) [45]	miR-30a-5p	NA	OP p’ts v.s. CTRLs	NA	NA	human blood sample in vitro: human MSC
Zhang et al. 2019 (b) [46]	miR-410	55	26 PM OP p’ts v.s. 29 CTRLs	all female	55.6 (OP), 55.1 (CTRL)	human blood sample in vitro: human & mice PBMC
Zhao et al. 2019 [47]	miR-21	96	48 OP p’ts v.s. 48 CTRLs	NA	NA	human blood sample in vitro: rats MSC
Zhou et al. 2019 [48]	miR let-7c	144	99 PM OP p’ts v.s. 45 premenopausal CTRLs	all female	40–65	human blood sample in vitro: human ASC
Du et al. 2020 [49]	miR-2861	40	20 OP p’ts v.s. 20 CTRLs	NA	NA	human blood sample in vitro: human MSC
Gao et al. 2020 [50]	miR-217	NA	OP p’ts v.s. CTRLs	NA	NA	human blood sample in vitro: human MSC
Ismail et al. 2020 [51]	miR-208a-3p miR-155-5p miR-637	140	70 OP p’ts v.s. 70 CTRLs	all female	61.3 (PM OP), 36.0 (premenopausal OP), 60.1 (PM CTRL), 34.0 (premenopausal CTRL)	human blood sample
Kaur et al. 2020 [52]	miR-300	60	30 OP p’ts v.s. 30 CTRLs	NA	NA	human blood sample In vitro: human & rat osteoblast cells In vivo: rat model
Lan et al. 2020 [53]	miR-429	60	30 OP p’ts v.s. 30 CTRLs	NA	NA	human blood sample in vitro: human ASC
Li et al. 2020 [54]	miR-483-5p	72	all have a hip frx 36 OP p’ts v.s. 36 non-OP CTRLs	all female	62 (OP), 59 (CTRL)	human blood sample in vitro: human PBMC
Mi et al. 2020 [55]	miR-194-5p	100	50 OP p’ts v.s. 50 non-OP CTRLs	NA	NA	human blood sample in vitro: mice MSC
Shuai et al. 2020 [56]	multiple	25 (discovery) 288 (training) 160(validation)	5 OP p’ts v.s. 10 LBM p’ts v.s. 10 CTRLs 86 OP p’ts v.s. 76 LBM p’ts v.s. 126 CTRLs 48 OP p’ts v.s. 56 LBM p’ts v.s. 56 CTRLs	NA 139: 149 73: 87	19–80	human blood sample
Sun et al. 2020 [57]	miR-19b	18 (discovery) 72 (validation)	6 OP p’ts v.s. 6 LBM p’ts v.s. 6 CTRLs 24 OP p’ts v.s. 24 LBM p’ts v.s. 24 CTRLs	3: 17 12: 60	73.1 (OP), 66.5 (LBM), 46.1 (CTRL)	human blood sample in vitro: human MSC and mice cell in vivo: mice model
Tang et al. 2020 [58]	multiple	36	all PM female 19 OP p’ts v.s. 17 CTRLs	all female	64.7 (OP) v.s. 58.1 (CTRL)	human blood sample human bone tissue in vitro: human osteoblast
Xu et al. 2020 [59]	miR-27a-3p	137	85 OP p’ts v.s. 52 CTRLs	all female	50–90	human blood sample In vitro: mice cell
Yin et al. 2020 [60]	miR-140-3p	60	30 PM OP p’ts v.s. 30 CTRLs	NA	NA	human blood sample in vitro: human PBMC & C2C12 cell
Yu et al. 2020 [61]	miR-137	51	30 OP p’ts with frx. v.s. 21 CTRLs	14: 37	60.8 (OP), 62 (CTRL)	human blood sample in vitro: human PBMC
Zarecki et al. 2020 [62]	multiple	116	all PM female 24 OP p’ts with frx. v.s. 17 OP p’ts with frx. under tx v.s. 35 LBM p’ts without frx. v.s. 40 CTRLs	all female	69.6 (OP with frx.), 69.6 (OP with frx. under treatment), 67.9 (PM LBM without frx.), 68.8 (CTRL)	human blood sample
Zhou et al. 2020 [63]	miR-1286	NA	OP p’ts v.s. CTRLs	NA	NA	human blood sample in vitro: human MSC

miR, microRNA or miRNA; OP, osteoporosis; LBM, low bone mass (i.e., osteopenia); CTRL, controls; p’t, patient; frx, fracture; PM, postmenopausal; ASC, adipocyte-derived stem cells; MSC, mesenchymal stem cells; PBMC, peripheral blood mesenchymal stem cells.

**Table 2:** Regulation of identified miRNAs in human blood samples in the setting of osteoporotic patients compared to controls.

**Osteoporotic Patients versus Controls**					
**Up-Regulated**			**Down-Regulated**		
**MiRNA**	**Correlation**	**Ref.**	**MiRNA**	**Correlation**	**Ref.**
miR-10b-5p		[24]	miR-19a-3p miR-21 miR-22-3p miR-27a-3p miR-122-5p miR-133b miR-203 miR-328-3p miR-518 miR-2861 miR-4516 miR-let-7g-5p		[32] [47] [16,24] [35] [27] [24] [31] [16,24] [24] [24] [27] [16,24]
miR-21		[24]			
miR-21-5p	linearly correlate with BMD	[22]			
miR-23		[24]			
miR-23b-3p	correlate with low BMD	[29]			
miR-24-3p	linearly correlate with BMD	[22]			
miR-27a-3p		[59]			
miR-30a-5p	may negatively correlate with XIXT	[45]			
miR-93-5p	linearly correlate with BMD	[22]			
miR-100		[24]			
miR-100-5p	linearly correlate with BMD	[22]			
miR-125b		[24]			
miR-125b-5p	linearly correlate with BMD	[22]			
miR-137		[61]			
miR-140-3p	correlate with low BMD	[29]			
miR-155-5p		[51]			
miR-194-5p		[55]			
miR-200a-3p		[41]			
miR-208a-3p		[51]			
miR-214		[20]			
miR-217	negatively correlate with RUNX2	[44]			
	may negatively correlate with TERC	[50]			
miR-300		[52]			
miR-365a-3		[34]			
miR-375		[36]			
miR-429		[53]			
miR-579-3p		[40]			
miR-637		[51]			
miR-1286		[63]			
**Osteoporotic Patients versus Osteopenia Patients versus Controls**					
**Up-Regulated**			**Down-Regulated**		
**MiRNA**	**Correlation**	**Ref.**	**MiRNA**	**Correlation**	**Ref.**
miR-30c-2-3p		[56]	miR-19b		[57]
miR-130b-3p	negatively correlate with BMD	[15]	miR-30b-5p	positively correlate with hip BMD	[18]
miR-151a-3p	negatively correlate with BMD	[15]	miR-103-3p	positively correlate with hip BMD	[18]
miR-151b	negatively correlate with BMD	[15]	miR-142-3p	positively correlate with hip BMD	[18]
miR-194-5p	negatively correlate with BMD	[15,56]	miR-199a-5p		[56]
miR-497-5p		[56]	miR-328-3p	positively correlate with hip BMD	[18]
miR-590-5p		[15]	miR-424-5p		[56]
miR-660-5p		[15]			
miR-877-3p		[56]			

aBMD, areal bone mineral density; RUNX2, runt-related transcription factor 2; TERC, telomerase RNA elements.

**Table 3:** Regulation of identified miRNAs in human blood samples in clinical setting regarding estrogen.

**Postmenopausal Osteoporotic Patients versus Controls**					
**Up-Regulated**			**Down-Regulated**		
**MiRNA**	**Correlation**	**Ref.**	**MiRNA**	**Correlation**	**Ref.**
miR-133a	negatively correlate with lumbar spine BMD	[25]	miR-28		[37]
			miR-101		[37]
miR-140-3p	negatively correlate with PTEN	[60]	miR-203		[28]
miR-338-3p		[39]	miR-373		[37]
miR-410	may negatively correlate with BMP-2	[46]			
miR-3065-5p		[39]			
**Postmenopausal Osteoporotic Patients versus Premenopausal Controls**					
**Up-Regulated**			**Down-Regulated**		
**MiRNA**	**Correlation**	**Ref.**	**MiRNA**	**Correlation**	**Ref.**
miR let-7c		[48]	miR-27a		[21]
**Postmenopausal Osteoporotic Patients versus Postmenopausal Non-Osteoporotic Controls**					
**Up-Regulated**			**Down-Regulated**		
**MiRNA**	**Correlation**	**Ref.**	**MiRNA**	**Correlation**	**Ref.**
miR-21	negatively correlate with BMD positively correlate with both RANKL and RANKL/OPG ratio	[42]	miR-21	positively correlate with hip and spine BMDs	[13]
miR-21-5p	negatively correlate with lumbar spine aBMD	[33]	miR-125b-5p	positively correlate with age	[33]
miR-23a-3p	positively correlate with TRAP5b	[33]	miR-330-3p		[58]
miR-133a	negatively correlate with hip and spine BMDs	[13]			
miR-135a-5p		[58]			
miR-144	positively correlate with Sfrp1	[43]			
miR-148a		[17]			
miR-181a-3p		[58]			
miR-188-3p		[58]			
miR-194-5p		[15]			
miR-576-3p		[58]			
miR-942		[58]			
**Postmenopausal Osteoporotic Patients with Fracture versus Postmenopausal Osteoporotic Patients without Fracture versus Postmenopausal Controls**					
**Up-Regulated**			**Down-Regulated**		
**MiRNA**	**Correlation**	**Ref.**	**MiRNA**	**Correlation**	**Ref.**
miR-19b-3p	positively correlate with serum levels of osteocalcin, ALP, and CTX	[62]	miR-21-5p	not found to correlate with BMD	[23]
miR-21-5p		[62]	miR-23a-3p	not found to correlate with BMD	[23]
miR-23a-3p		[62]	miR-29a-3p	not found to correlate with BMD	[23]
miR-124-3p	not found to correlate with BMD	[23]			
miR-152-3p		[62]			
miR-335-5p		[62]			
miR-375		[62]			
miR-532-3p	positively correlate with ALP	[62]			
miR-2861	not found to correlate with BMD	[23]			

PTEN, phosphatase and tensin homolog; Sfrp1, secreted frizzled related protein 1; TRAP5b, tartrate-resistant acid phosphatase 5b; ALP, alkaline phosphatase; CTX, C-terminal telopeptide.

**Table 4:** Regulation of identified miRNAs in human blood samples in clinical setting regarding fracture.

**Osteoporotic Patients with Low-Traumatic Fracture versus Controls**					
**Up-Regulated**			**Down-Regulated**		
**MiRNA**	**Correlation**	**Ref.**	**MiRNA**	**Correlation**	**Ref.**
miR-152–3p		[19]	miR-19a-3p		[19]
miR-335–5p		[19]	miR-19b-3p	correlated with lumbar spine aBMD	[19]
			miR-30e-5p		[19]
			miR-140–5p		[19]
			miR-324–3p	correlated with lumbar spine aBMD	[19]
			miR-550a-3p		[19]
**Osteoporotic Patients with Fracture versus Non-Osteoporotic Controls with Fracture**					
**Up-Regulated**			**Down-Regulated**		
**MiRNA**	**Correlation**	**Ref.**	**MiRNA**	**Correlation**	**Ref.**
miR-21		[14]	miR-144-3p		[37]
miR-23a		[14]			
miR-24		[14]			
miR-24-3p		[30]			
miR-25		[14]			
miR-27a-3p		[30]			
miR-93		[14]			
miR-100		[14,30]			
miR-122a		[14,30]			
miR-124a		[14]			
miR-125b		[14,30]			
miR-148a		[14]			
miR-363-3p		[38]			
miR-483-5p	may negatively correlate with IGF2	[54]			

IGF2, insulin-like growth factor-2.

**Table 5:** Regulation of identified miRNAs in human blood samples in clinical setting regarding advanced age.

Elderly Osteoporotic Patients versus Elderly Controls					
Up-Regulated			Down-Regulated		
MiRNA	Correlation	Ref.	MiRNA	Correlation	Ref.
miR-96		[26]			
miR-107		[26]			

**Table 6:** ROC analysis on high-potential miRNAs as biomarkers for osteoporosis; NA, not available.

Study Setting	MiRNA	Area under Curve (AUC)	Sensitivity	Specificity	Reference
Osteoporotic patients v.s. controls	miR-10b-5p	0.87	NA	NA	[24]
	miR-23b-3p	0.69	NA	NA	[29]
	miR-100	0.89	NA	NA	[24]
	miR-140-3p	0.92	NA	NA	[29]
	miR-300	0.969	NA	NA	[52]
	miR-328-3p	0.87	NA	NA	[24]
	miR-4516	0.727	71%	62%	[27]
	let-7g-5p	0.89	NA	NA	[24]
Premenopausal osteoporotic patients v.s. controls	miR-155-5p	0.9	94.29%	77.14%	[51]
	miR-208a-3p	0.816	77.14%	82.86%	[51]
Postmenopausal osteoporotic patients v.s. controls	miR-135a-5p	0.759	NA	NA	[58]
	miR-155-5p	0.828	80%	80%	[51]
	miR-181a-3p	0.817	NA	NA	[58]
	miR-188-3p	0.889	NA	NA	[58]
	miR-208a-3p	0.851	80%	82.86%	[51]
	miR-338-3p	0.74	NA	NA	[39]
	miR-576-3p	0.751	NA	NA	[58]
	miR-637	0.814	77.14%	85.71%	[51]
	miR-942-3p	0.678	NA	NA	[58]
	miR-3065-5p	0.87	NA	NA	[39]
Postmenopausal osteoporotic patients with fracture. v.s.	miR-21-5p	0.66	66%	71%	[23]
postmenopausal osteoporotic patients without fracture v.s.					
postmenopausal controls					
Osteoporotic patients with fracture v.s. non-osteoporotic controls with fracture	miR-122a	0.77	74.14%	72.14%	[14]

**Table 7:** Mechanisms of the identified miRNAs in human blood sample for osteoblastogenesis supported by in vitro and/or in vivo experiments.

Involved Pathways	MiRNA	Target	Effect to Osteoblasto-Genesis	In Vitro Evidence		In Vivo Evidence			Ref.
				Regulation of Target Gene Confirmed by miRNA Mimics or Inhibitor Transfection	Effect of miRNA Altered by Overexpression, Knockdown or Silence of the Target Gene	By DXA	By micro-CT		
Wnt	miR-23b-3p	MRC2	inhibition	v (wild type v.s. mutant UTR)	v	Mice model			[69]
						v	v		
	miR-27a	Mef2c	promotion	v (wild type v.s. mutant UTR)	v	Mice model			[21]
						v	v		
	miR-144	Sfrp1	promotion	v (wild type v.s. mutant UTR)	v				[43]
	miR-194-5p	Wnt 5a	inhibition	v (wild type v.s. mutant UTR)		Mice model			[55]
							v		
	miR-203	DKK1	promotion	v (wild type v.s. mutant UTR)		Rat model			[28,31]
							v		
	miR-429	SCD-1	inhibition	v (wild type v.s. mutant UTR)	v				[53]
	miR-579-3p	Sirt	inhibition	v (wild type v.s. mutant UTR)	v				[40]
	miR let-7c	SCD-1	inhibition	v (wild type v.s. mutant UTR)	v				[48]
TGF-β	miR-300	Smad	inhibition	v		Rat model			[52]
						v	v		
BMP	miR-410	BMP-2	inhibition	v (wild type v.s. mutant UTR)					[46]
Common regulatory factors	miR-30a-5p	RUNX2	inhibition	v (wild type v.s. mutant UTR)	v				[45]
	miR-217	RUNX2		v (wild type v.s. mutant UTR)	v				[50] [44]
	miR-365a-3p	RUNX2		v (wild type v.s. mutant UTR)	v				[34]
	miR-375	RUNX2		v (wild type v.s. mutant UTR)	v				[36]
	miR-27a-3p *	osterix	inhibition	v (wild type v.s. mutant UTR)	v				[59]
	miR-96	osterix		v (wild type v.s. mutant UTR)	v	Mice model			[26]
	
	miR-637	osterix		v (wild type v.s. mutant UTR)					[70]
Others	miR-19a-3p	HDAC4	promotion	v (wild type v.s. mutant UTR)	v				[32]
	miR-19b	PTEN	promotion	v (wild type v.s. mutant UTR)		Mice model			[57]
							v		
	miR-27a-3p *	ATF3	promotion	v (wild type v.s. mutant UTR)	v				[35]
	miR-200a-3p	glutaminase	inhibition	v (wild type v.s. mutant UTR)	v				[41]
	miR-208a-3p	ACVR1	inhibition	v (wild type v.s. mutant UTR)		Mice model			[71]
								v	
	miR-338-3p	PCSK5	inhibition	v (wild type v.s. mutant UTR)	v				[72]

* It is noteworthy that miR-27a-3p is found to target both osterix and ATF3 gene with opposite effect on osteoblastogenesis. In Fu et al. 2019, human MSCs transfected with miR-27a-3p mimics have higher activity of osteogenic differentiation [35]; whereas MC3T3-E1 cells transfected with miR-27a-3p mimics have decreased expression of osteoblast marker genes in Xu et al. 2020 [59]. MRC2, mannose receptor C type 2; Mef2c, myocyte enhancer factor 2c; ATF3, activating transcription factor 3; PCSK5, proprotein convertase subtilisin/kexin type 5

**Table 8:** Mechanisms of the identified miRNAs in human blood sample for osteoclastogenesis supported by in vitro and/or in vivo experiments.

Involved Pathways	MiRNA	Target	Effect to Osteoclasto- Genesis	In Vitro Evidence		In Vivo Evidence		Ref.
				Regulation of Target Gene Confirmed by miRNA Mimics or Inhibitor Transfection	Effect of miRNA Altered by Overexpression, Knockdown or Silence of the Target Gene	By DXA	By Micro-CT	
RANK	miR-133a		promotion	v		Rat model		[25]
							v	
	miR-144-3p	SMAD4 RANK	inhibition	v (wild type v.s. mutant UTR)				[30]
	miR-338-3p *	MafB	promotion	v (wild type v.s. mutant UTR)	v			[86]
PTEN/PI3K/AKT signaling pathway	miR-140-3p	PTEN	promotion	v (wild type v.s. mutant UTR)	v			[60]
	miR-363-3p	PTEN		v (wild type v.s. mutant UTR)	v			[38]
	miR-2861	AKT2	inhibition	v				[49]
Others	miR-155-5p	MITF	inhibition	v				[87]
	miR-338-3p *	IKKβ gene	inhibition	v (wild type v.s. mutant UTR)				[88]

* It is noteworthy that miR-338-3p is found to target both MafB and IKKβ genes with conflicting effect on osteoclastogenesis. In Sun et al. 2019, RAW264.7 cells transfected with miR-338-3p mimics have higher activity of osteoclast differentiation [86], whereas RAW264.7 cells transfected with miR-338-3p mimics have decreased expression levels of important proteins for osteoclastosis in Niu et al. 2019 [88].

**Table 9:** Recommended miRNAs candidates in diagnostic panels and as therapeutic agents in osteoporosis.

MiRNA	Clinical Setting	Mechanism (Target)	Conflicting Results
miR-23b-3p	OP p’ts v.s. CTRLs	Inhibition of osteoblastogenesis (MRC2)	
miR-140-3p	OP p’ts v.s. CTRLs	Promotion of osteoclastogenesis (PTEN)	
miR-300	OP p’ts v.s. CTRLs	Inhibition of osteoblastogenesis (Smad)	
miR-155-5p	PM OP p’ts v.s. CTRLs	Inhibition of osteoclastogenesis (MITF)	
miR-208a-3p	PM OP p’ts v.s. CTRLs	Inhibition of osteoblastogenesis (ACVR1)	
miR-338-3p	PM OP p’ts v.s. CTRLs	Inhibition of osteoblastogenesis (PCSK5) Conflicting results on osteoclastogenesis (MafB, IKKβ)	v
miR-637	PM OP p’ts v.s. CTRLs	Inhibition of osteoblastogenesis (osterix)	

miR, microRNA or miRNA; OP, osteoporosis; PM, postmenopausal; p’t, patient; CTRL, controls.
